# Social isolation, loneliness and low dietary micronutrient intake amongst older people in England

**DOI:** 10.1093/ageing/afae223

**Published:** 2024-10-16

**Authors:** Andrew Steptoe, Hoi Lam Fong, Camille Lassale

**Affiliations:** Department of Behavioural Science and Health, University College London, London, UK; Department of Behavioural Science and Health, University College London, London, UK; ISGlobal, Barcelona, Spain; Universitat Pompeu Fabra, Barcelona, Spain; CIBER of Physiopathology of Obesity and Nutrition, Institute of Health Carlos III, Madrid, Spain

**Keywords:** nutrition, social isolation, diet, loneliness, older people

## Abstract

**Background:**

Social isolation and loneliness are associated with increased risk of adverse health outcomes at older ages. This study evaluated whether isolation and loneliness are related to inadequate intake of micronutrients in the diet.

**Methods:**

We tested associations between social isolation and loneliness and dietary micronutrient intake 2 years later in 3713 men and women (mean age 68.26, standard deviation 7.81 years) who completed two online 24-h dietary recalls. Associations of isolation and loneliness with intake of nine minerals and vitamins that fell below national recommendations were tested using logistic regressions, adjusting for age, gender, ethnicity, education, marital status, smoking and physical activity and total energy intake.

**Results:**

The prevalence of low dietary intake varied markedly across micronutrients. Social isolation (1-point increase in a score ranging 0–5) was associated with increased odds (adjusted for covariates) of low intake of magnesium [odds ratio (OR) 1.153, 95% confidence interval (CI) 1.037–1.282, *P* = .009], potassium (OR 1.201, 95% CI 1.087–1.327, *P* < .001), vitamin B6 (OR 1.263, 95% CI 1.110–1.438, *P* < .001), folate (OR 1.211, 95% CI 1.093–1.341, *P* < .001) and vitamin C (OR 1.238, 95% CI 1.098–1.394, *P* < .001). These associations remained unchanged when food insecurity and impaired activities of daily living were taken into account. By contrast, loneliness was not related to the inadequate intake of any micronutrient.

**Conclusions:**

Low intake of micronutrients increases risk of age-related health problems. Attention to the dietary quality of older people with limited social contacts and little involvement in community activities might enhance health outcomes.

## Key points

- Existing studies show that social isolation and loneliness are correlated with low fruit and vegetable consumption, and with a lower overall diet quality.
- No previous study has focused on micronutrient intake.
- In older adults in the UK, we found associations between social isolation and suboptimal intake of micronutrients: magnesium, potassium, vitamin B6, folate and vitamin C, adjusting for confounders.
- No association was found between loneliness and micronutrient intake.
- Low intake of micronutrients may be a mechanism through which social isolation increases risk of age-related health problems.

## Introduction

Adequate dietary micronutrient intake is essential to healthy ageing, and deficiencies increase risk of impaired function across multiple systems ([1]). Calcium, vitamin D, magnesium and potassium are implicated in bone health, whilst reduced intake of folate, B vitamins and vitamin E is associated with risk of cardiovascular disease and cognitive decline [2–4]. Iron is important for the maintenance of physical capability, and low intake reduces work capacity and may also increase dementia risk [5]. Vitamins C and D have multiple roles in maintaining health. There is substantial evidence that dietary micronutrient deficiencies are common amongst older men and women in high income countries. A review of multiple cross-sectional and longitudinal studies of people aged 65 and older concluded that 20%–35% were consuming lower than recommended levels of folate, vitamins B6, C and E, with even higher proportions not eating adequate amounts of calcium and magnesium [6]. Analyses of bloods from population cohorts indicate iron deficiencies in 8%–11% of older people [7, 8].

Understanding the factors associated with micronutrient deficiencies in the diet will help identify vulnerable sectors of the population. Advancing age, lower socioeconomic status and depressed mood are relevant [7, 9, 10]. A further possibility is that social isolation and loneliness increase risk of dietary deficiencies. Social isolation refers to the combination of living alone, having infrequent of social contacts and little engagement in community activities, whilst loneliness is a subjective distressing feeling of isolation and lack of companionship [11]. Social isolation and loneliness have been linked in large-scale longitudinal studies with multiple adverse health outcomes including depression, dementia, coronary heart disease, disability and mortality [11–13]. They have been recognised as important public health issues, particularly amongst older people [14, 15].

Social isolation and loneliness are thought to affect physical health through a combination of biological and behavioural processes. The biological pathways include heightened inflammatory, autonomic and neuroendocrine activation [16, 17]. Behavioural and lifestyle factors include unhealthy behaviours, particularly smoking, physical inactivity and poor adherence to medical advice, all of which have been linked to both isolation and loneliness [18–21]. Research on diet has primarily focused on fruit and vegetable consumption, intake of nutrient-poor energy-dense items (e.g. sugar sweetened beverages), and on indices of overall diet quality. The evidence-base summarised in a 2023 review [22] indicates more consistent links between social isolation and fruit and vegetable intake than for loneliness and fruit and vegetables [23–26]. But no large-scale studies have tested whether social isolation and loneliness are related to micronutrient intake.

Our aim was to assess whether social isolation and loneliness were associated with 9 micronutrient intakes (calcium, iron, magnesium, potassium, and vitamins C, B6, B12, folate, and vitamin E) below nationally recommended levels amongst people aged 50 and older. We also tested whether food insecurity, impaired activities of daily living and the use of dietary supplements modified relationships between social isolation, loneliness and low micronutrient intake.

## Method

The English Longitudinal Study of Ageing (ELSA) involves a representative sample of men and women aged 50 and older living in the community in England [27]. The study started in 2002/03, with 12 099 participants ranging in age from 50 to >90 years, and new respondents are periodically added to maintain the full age range. The primary forms of data collection are a computer-assisted personal interview carried out in participants’ homes supplemented with a self-completion questionnaire, typically carried out every 2 years, with additional sets of assessments on specific issues. The online nutritional assessment was included in Wave 9 (2018/19), and was completed by 5017. The social isolation and loneliness measures were obtained 2 years earlier in Wave 8 (2016/17). The number of individuals with data on nutrition, social isolation and loneliness was 3988. Data were missing on one or more covariates for 217 individuals, resulting in a sample of 3771.

### Dietary assessment

Diet was assessed on two non-consecutive days using the Oxford WebQ, an online 24 h recall tool implemented in large-scale studies in the UK (UK Biobank, Million Women Study) [28]. The questionnaire contains >200 items that are grouped into 21 food and beverage categories, and respondents indicate what items they have consumed and in what quantities, with images of portion sizes to improve accuracy of reporting. These assessments have been validated against nutritional biomarkers and are acceptable to older respondents [29, 30]. A profile of the intake of 21 separate nutrients, including total energy intake (kcal), macro and micronutrients is calculated by the WebQ team through multiplication of amounts consumed by the nutrient contents specified in standard UK food composition tables [31]. Participants were also questioned about their use of mineral and vitamin supplements. We excluded from the analysis individuals whose energy intakes were implausibly high or low, applying thresholds of <800 or >4200 kcal/day for men and <600 or >3500 for women [32], resulting in the exclusion of 58 individuals. The final analytic sample was 3713 (1689 men and 2024 women). A complete case analysis was carried out in view of the small number of cases with missing data on covariates [33].

### Social isolation and loneliness

Social isolation and loneliness were assessed in Wave 8 (2016/17) with well-established measures [34, 35]. A composite index, including whether or not the individual lived alone, frequency of social contacts with children and other relatives outside the household and with friends, and whether or not they participated in organisations and clubs, was used to measure social isolation [34, 36]. The total score ranged from 0–5, with higher scores indicating greater isolation. This index has been shown in prospective studies to be associated with unhealthy lifestyles, adverse biomarker profiles, disability and mortality [23, 36–38]. Loneliness was measured with the short University of California, Los Angeles scale, a three-item questionnaire (e.g. ‘how often do you feel you lack companionship’), each of which is rated on a three category scale [35]. Scores range from 3–9 with higher ratings indicating greater loneliness.

### Covariates

Covariates were assessed in Wave 9 (2018/19) at the same time as nutrition. Age was modelled as a continuous variable. Gender was divided into male and female, and ethnicity into White and non-White. Education was measured as the age at which the participant completed their schooling, ranging from 14 to ≥19 years. Participants were categorised on marital status as currently living with a partner or not. Current smoking was assessed by self-report, whilst physical activity was measured by enquiring about frequency of participation in vigorous (e.g. jogging, aerobics), moderate (e.g. gardening) and light (e.g. home repairs) sports or activities. A five-level measure was constructed as detailed and validated elsewhere, ranging from sedentary to frequent vigorous activity [39]. Food insecurity was assessed in 3625 participants in a separate section of the study by asking respondents whether over the past 12 months (i) the food they bought did not last and they did not have enough money to get more; and (ii) they could not afford to eat balanced meals. Response options were ‘never true’, ‘sometimes true’, and ‘often true’. Individuals who answered sometimes or often true on either item were classified as experiencing food insecurity. Other factors such as depressive symptoms and health status were not included as covariates because the rationale underlying the analyses was that nutrition deficiencies may act as mediators of social isolation and loneliness with adverse health outcomes, so adjusting for these factors increase risk of collider bias.

### Statistical analysis

Participants were divided into those with sufficient or low dietary intake of micronutrients, based on the criteria described in the UK’s Scientific Advisory Committee for Nutrition (SACN) statement on nutrition and older adults living in the community [40]. Multivariable logistic regression models were used to estimate the odds of low dietary intake of each micronutrient (outcome) in relation to social isolation (exposure 1) or loneliness (exposure 2). For each exposure, two models were tested: model 1 adjusted for age, gender and total energy intake; and model 2 additionally adjusted for ethnicity, education, marital status, smoking and physical activity. Results are presented as adjusted odds ratios (OR) with 95% confidence intervals (CI) per unit increase in the social isolation or loneliness scale. A separate set of analyses including food insecurity as a covariate was carried out on a reduced sample size. In supplementary analyses, we stratified the sample into younger (<65 years) and older (≥65 years) participants. We also repeated the analyses amongst people who were not taking dietary supplements relevant to each micronutrient. Finally, we conjectured that functional difficulties might restrict food choice and also contribute to social isolation/loneliness. We, therefore, measured impairments in a series of basic (e.g. difficulty cutting up food) and more complex activities of daily living (e.g. preparing a hot meal, shopping for groceries), and added the presence of impairment as a covariate in supplementary analysis.

## Results

The study sample comprised 2024 women and 1689 men, with an average age of 68.26 ± (SD) 7.81 years (see Table 1). Slightly less than a third of the participants (*N* = 1170) were aged <65, with 2343 (68.5%) being aged 65 and older. The large majority (97.7%) were White, and the mean age of completing schooling was 16.84 ± 1.70 years, with 1041 (28.0%) continuing their education beyond age 19. The majority (79.3%) were partnered, only 6% were smokers, and 17.2% reported impaired activities of daily living. Food insecurity was experienced by 156 (4.3%) participants. The full range of social isolation and loneliness ratings from lowest to highest possible values was represented, and social isolation and loneliness were weakly correlated (*r* = 0.112, *P* < .001).

**Table 1:** Characteristics of the participants.

	Complete sample Mean ± SD or N (%)	Age < 65 years Mean ± SD or N (%)	Age ≥ 65 years Mean ± SD or N (%)
Gender Women Men	2024 (54.5%) 1689 (45.5%)	709 (60.6%) 461 (39.4%)	1315 (51.7%) 1228 (48.3%)
Age, years	68.26 ± 7.81	59.70 ± 3.95	72.20 ± 5.71
Ethnicity White Non-white	3627 (97.7%) 86 (2.3%)	1123 (96.0%) 47 (4.0%)	2504 (98.5%) 39 (1.5%)
Education, years	16.84 ± 1.70	17.11 ± 1.59	16.72 ± 1.73
Marital status Married/partnered Not married	2945 (79.3%) 768 (20.7%)	972 (83.1%) 198 (16.9%)	1973 (77.6%) 570 (22.4%)
Current smoking	222 (6.0%)	93 (92.1%)	129 (5.1%)
Physical activity index	2.39 ± 1.27	2.62 ± 1.25	2.29 ± 1.26
Impaired activities of daily living	637 (17.2%)	156 (13.3%)	481 (18.9%)
Food insecurity (*n* = 3625)	156 (4.3%)	71 (6.3%)	85 (3.4%)
Social isolation	0.79 ± 0.85	0.83 ± 0.85	0.77 ± 0.85
Loneliness	3.89 ± 1.36	3.95 ± 1.41	3.83 ± 1.30

Average daily energy intake was estimated as 2009 ± 567 Kcal. As expected, mean energy intake greater amongst men (2184 ± 591) than women (1865 ± 501). After adjustment for age and gender, total energy intake was unrelated to social isolation (*r* = −0.015, *P* = .35) but was weakly negatively associated with loneliness (*r* = −0.042, *P* = .010).

Table 2 summarises estimates of dietary micronutrient intake. For seven micronutrients, the prevalence of inadequate intake was >10%, ranging from 10.9% for vitamin C to 53.2% for potassium. The proportions of respondents having inadequate dietary intake of vitamins B12 and E were low. The number of participants who reported taking dietary supplements was moderate, ranging from 4.2% for iron to 13.7% for vitamin C.

**Table 2:** Dietary micronutrient intake.

Micronutrient	Estimated daily intake Mean ± SD	Reference dietary intake threshold	Sufficient N (%)	Low N (%)	Supplements N (%)
Calcium (mg/day)	919.2 ± 317.2	700 mg/day	2774 (74.7%)	939 (25.3%)	270 (7.2%)
Iron (mg/day)	12.7 ± 4.11	8.7 mg/day	3112 (83.8%)	601 (16.2%)	157 (4.2%)
Magnesium (mg/day)	340.2 ± 99.4 306.4 ± 92.1	300 mg/day (men) 270 mg/day (women)	2355 (63.4%)	1358 (36.4%)	388 (10.4%)
Potassium (mg/day)	3511.0 ± 1065.5	3500 mg/day	1739 (46.8%)	1974 (53.2%)	324 (8.7%)
Vitamin B6 (mg/day)	2.17 ± 0.71 1.97 ± 0.67	1.0 mg/day (men) 0.8 mg/day (women)	3294 (88.7%)	419 (11.3%)	376 (10.2%)
Folate (μg/day)	282.4 ± 109.6	200 μg/day	2884 (77.7%)	829 (22.3%)	367 (9.9%)
Vitamin B12 (μg/day)	6.31 ± 4.00	1.5 (μg/day)	3580 (96.4%)	133 (3.6%)	409 (11.0%)
Vitamin C (mg/day)	132.1 ± 94.5	40 mg/day	3307 (89.1%)	406 (10.9%)	508 (13.7%)
Vitamin E (mg/day)	8.78 ± 4.04 8.73 ± 3.95	4.0 mg/day (men) 3.0 mg/day (women)	3500 (94.3%)	213 (5.7%)	351 (9.4%)

We found that social isolation was associated with a higher risk of low intake for five of the nine micronutrients evaluated, namely magnesium, potassium, folate, vitamin B6 and vitamin C, with a marginal relationship for iron (Table 3). Associations were apparent not only in the basic models adjusting for age, gender and total energy intake, but also in multiply adjusted models. The odds ratios ranged from 1.153 (95% CI 1.037–1.282) for magnesium to 1.238 (95% CI 1.098–1.394) for vitamin C. The association for iron was (1.140, 95% CI 1.002–1.298), whilst effects for calcium and vitamins B12 and E were not significant.

**Table 3:** Odds of low dietary micronutrient intake associated with social isolation and loneliness (*n* = 3713).

	Social isolation				Loneliness			
	Adjusted for age, gender and total energy intake		Fully adjusted^a^		Adjusted for age, gender and total energy intake		Fully adjusted^a^	
	OR (95%CI)	P	OR (95%CI)	P	OR (95%CI)	P	OR (95%CI)	P
Calcium	1.090 (0.985–1.206)	0.097	1.060 (0.956–1.176)	0.27	0.982 (0.923–1.045)	0.57	0.956 (0.895–1.021)	0.18
Iron	1.224 (1.079–1.389)	0.002	1.140 (1.002–1.298)	0.047	1.093 (1.014–1.178)	0.020	1.035 (0.956–1.120)	0.39
Magnesium	1.254 (1.131–1.390)	<0.0001	1.153 (1.037–1.282)	0.009	1.107 (1.040–1.178)	0.002	1.066 (0.997–1.140)	0.060
Potassium	1.255 (1.138–1.384)	<0.0001	1.201 (1.087–1.327)	<0.001	1.111 (1.045–1.180)	<0.001	1.071 (1.004–1.142)	0.037
Vitamin B6	1.299 (1.145–1.474)	<0.0001	1.263 (1.110–1.438)	<0.001	1.071 (0.991–1.157)	0.084	1.040 (0.958–1.129)	0.35
Folate	1.278 (1.156–1.423)	<0.0001	1.211 (1.093–1.341)	<0.001	1.080 (1.025–1.157)	0.005	1.057 (0.991–1.126)	0.090
Vitamin B12	0.948 (0.768–1.169)	0.62	0.911 (0.732–1.133)	0.40	1.044 (0.928–1.175)	0.48	0.991 (0.872–1.126)	0.89
Vitamin C	1.348 (1.202–1.514)	<0.0001	1.238 (1.098–1.394)	<0.001	1.117 (1.041–1.198)	0.002	1.056 (0.979–1.139)	0.16
Vitamin E	1.203 (1.017–1.423)	0.031	1.111 (0.933–1.324)	0.24	1.152 (1.040–1.278)	0.007	1.102 (0.986–1.232)	0.087

a Adjusted for age, gender, ethnicity, education, total energy intake, marital status, smoking and physical activity.

By contrast, there were few associations between loneliness and micronutrients. Although intake of iron, magnesium, potassium, folate, vitamin C and vitamin E were related to loneliness in basic models adjusted for age, gender and total energy intake, none of the associations remained following adjustment for ethnicity, education, marital status, smoking and physical activity (Table 3).

A second set of analyses were carried out with inclusion of food insecurity as an additional covariate (Table 4): results were similar to the primary analyses for social isolation, and a weak association between loneliness and potassium insufficient intake was evident.

**Table 4:** Odds of low micronutrient intake associated with social isolation and loneliness—additional adjustment for food insecurity (*n* = 3625).

	Social isolation				Loneliness			
	Adjusted for age, gender and total energy intake		Fully adjusted^a^		Adjusted for age, gender and total energy intake		Fully adjusted^a^	
	OR (95%CI)	P	OR (95%CI)	P	OR (95%CI)	P	OR (95%CI)	P
Calcium	1.090 (0.983–1.209)	0.10	1.059 (0.953–1.176)	0.29	0.984 (0.923–1.049)	0.62	0.954 (0.891–1.021)	0.18
Iron	1.220 (1.073–1.387)	0.002	1.141 (1.000–1.302)	0.049	1.072 (0.993–1.158)	0.075	1.015 (0.934–1.102)	0.73
Magnesium	1.255 (1.131–1.394)	<0.0001	1.156 (1.038–1.288)	0.008	1.103 (1.035–1.176)	0.003	1.064 (0.993–1.140)	0.080
Potassium	1.262 (1.143–1.394)	<0.0001	1.208 (1.092–1.337)	<0.001	1.113 (1.046–1.184)	<0.001	1.075 (1.007–1.148)	0.030
Vitamin B6	1.280 (1.127–1.456)	<0.0001	1.246 (1.093–1.421)	0.001	1.048 (0.967–1.135)	0.25	1.015 (0.931–1.106)	0.74
Folate	1.271 (1.148–1.407)	<0.0001	1.212 (1.092–1.344)	<0.001	1.085 (1.020–1.154)	0.009	1.060 (0.993–1.132)	0.082
Vitamin B12	0.945 (0.764–1.169)	0.60	0.914 (0.733–1.140)	0.42	1.029 (0.910–1.162)	0.65	0.983 (0.861–1.122)	0.80
Vitamin C	1.352 (1.203–1.520)	<0.0001	1.245 (1.103–1.406)	<0.001	1.125 (1.047–1.208)	0.001	1.065 (0.985–1.151)	0.11
Vitamin E	1.203 (1.015–1.427)	0.033	1.116 (0.935–1.331)	0.23	1.151 (1.035–1.279)	0.009	1.115 (0.994–1.251)	0.064

a Adjusted for age, gender, ethnicity, education, total energy intake, marital status, smoking, physical activity and food insecurity.

### Supplementary analyses

Division of the study sample into those aged above and <65 years showed similar levels of micronutrient intake and proportion of people taking supplements in older and younger groups (Supplementary Tables S1 and S2). In subgroup analyses (Supplementary Tables S3 and S4), the smaller sample sizes reduced statistical power, and confidence intervals overlapped, but there were some indications for more robust associations in the older than younger categories. In the analyses of loneliness, an association emerged with vitamin C intake in the fully adjusted model in the younger group only (OR = 1.157, 95% CI 1.029–1.300).

To identify inadequate micronutrient intake more clearly, we conducted the analyses excluding participants who reported taking dietary supplements. These showed very similar results to the main findings (Supplementary Table S5). As before, greater social isolation was associated with increased risk of deficiency in magnesium, potassium, vitamin B6, folate and vitamin C in the diet, and there were no relationships between loneliness and micronutrient intake. Finally, a further sensitivity analysis added impaired activities of daily living as a covariate, but this did not modify the patterns of results (Supplementary Table S6).

## Discussion

This study of dietary micronutrient intake amongst older men and women in England demonstrated that consumption was below recommended levels in a substantial number of participants. Risk of low intake was associated with social isolation for five micronutrients—magnesium, potassium, folate, vitamins B6 and C—after adjustment for age, gender, ethnicity, total energy intake, education, marital status, smoking and physical activity. Estimates ranged from a 15%–23% increased odds of deficiency per unit increase on the social isolation scale. The relationship was somewhat more robust for older (≥65 years) than younger (50–64 years) participants, and were unchanged when dietary supplements, and impaired activities of daily living were taken into account. The pattern of results was the same when food insecurity was included as an additional covariate in a subset of respondents.

Striking numbers of respondents in this study had levels of vitamin and mineral intake below recommended levels, potentially placing them at increased risk for a range of adverse health outcomes. Whilst a large majority reported consuming sufficient vitamins B12 and E, ~10%–20% consumed insufficient iron and vitamins C and B6, and even higher proportions had low calcium, magnesium, potassium and folate in their diets. A comparison of the prevalence of inadequate micronutrients with that detailed in a systematic review of studies involving older community-dwelling people shows similar levels for folate and iron, but a lower proportion in the present study for calcium, magnesium and vitamins B6 and C [6]. These variations relate in part to different criteria for adequate intakes; the thresholds used by ter Borg *et al.* were based on the Nordic Nutrition or US Institute of Medicine recommendations, whereas we used the UK SACN. Methods of dietary data collection are also relevant, with different studies using 24 h dietary recall, food frequency questionnaires and weighed records. The most relevant comparison for the present study is with UK Biobank because the same online 24 h recall instrument was used. The average intakes of micronutrients recorded in ELSA compare well with those recorded in UK Biobank [32].

The most important finding of these analyses is that social isolation is associated with low levels of intake of five micronutrients that are essential for health. We took account of a number of factors that might be responsible for these relationships. For example, micronutrient intake is likely to be lower amongst people who are malnourished and eat fewer calories. It is also possible that people who eat an unhealthy diet with low micronutrient intake might have a high energy intake (consuming energy-dense nutrient-poor foods, such as fast and ultra-processed food). Adjusting for energy intake is a common strategy in nutritional epidemiology to correct for measurement error in dietary assessment tools. In this study, isolation was negatively correlated with total energy intake, but the link with micronutrients was present after this was taken into account. Similarly, marital status, educational attainment, smoking and physical activity are relevant, but the associations remained robust after these factors were included as covariates. Nor could associations be accounted for by food insecurity, since relationships remained unchanged when participants’ reports of not having enough money to eat in a healthy way were taken into account. Difficulty in accessing shops to buy healthy food is unlikely to be an issue, since including limitations in mobility and activities of daily living did not modify the results.

It is notable that whilst social isolation was consistently associated with risk of dietary micronutrient deficiency, loneliness was not. Some other population studies of fruit and vegetable consumption have shown a similar pattern, with links observed with isolation but not loneliness when analyses are adjusted for relevant covariates [23, 26, 41]. One explanation is that deficits in micronutrient intake may be more strongly affected by the material and practical consequences of impoverished social connections than by emotional components. Older individuals with less frequent social contacts may not have people around them who can provide information on healthy diet and encourage simple recommendations. Older people favour diets that are familiar rather than adventurous [42], and individuals living alone may enjoy less dietary variety and less healthy dietary intake [43].

We recorded very few differences in the relationships between isolation and micronutrient intake in late middle-aged/young older people (<65 years) compared with older respondents (≥ 65 years). Although some associations appeared stronger amongst older participants (e.g. magnesium, folate and B6), the differences were small and the smaller number of younger versus older respondents reduced statistical power. It is possible that more substantial differences might emerge in very old study samples [44], but numbers in the present cohort were insufficient to create finer age stratification.

Social isolation and loneliness were measured 2 years before diets, so were assessed before the outcome; nevertheless, this is an observational study and causality cannot be determined. We adjusted statistically for demographic factors, total energy intake, sociodemographic factors and health behaviours. A limitation is that dietary assessments were based on two 24 h recalls; although this method is appropriate for large-scale population samples, it has inherent measurement error [45]. Moreover, dietary intake might not be directly linked to circulating levels of vitamin or minerals. Accompanying dietary assessments with blood sampling would provide a more comprehensive evaluation of links between social isolation and micronutrient deficiencies. The measures of dietary supplements were limited in that quantities of supplementation could not be established.

Nonetheless, this study does establish important relationships between inadequate dietary intake of minerals and vitamins and social isolation amongst older people. It, therefore, provides an evidence base for targeting dietary quality amongst men and women with limited social contacts and little involvement with community and social activities. Based on the pattern of deficiencies (magnesium, potassium, folate and vitamins B6 and C), the food sources lacking are mostly nutrient-rich plant-based foods (fruit, vegetables—such as dark leafy greens, legumes, nuts, seeds and whole grain) and fish, rather than other animal foods (dairy, egg, meat), which are major sources of calcium, iron and vitamin B12. Attention both to the adequacy of nutritional intake and to foods that enhance micronutrient content would help redress these deficits and help promote healthier ageing.

## Supplementary Material

aa-24-0959-File002_afae223
