# Supplementary material for: Social isolation, loneliness and low dietary micronutrient intake amongst older people in England
Source: Age Ageing. 2024 Oct 16;53(10):afae223. doi: 10.1093/ageing/afae223 (PMC11479707; doi:10.1093/ageing/afae223)
Supplement: aa-24-0959-File002_afae223 [file aa-24-0959-file002_afae223.pdf]

Social isolation, loneliness, and low dietary micronutrient intake among older people in England

Supplementary Material

Table S1 Dietary micronutrient intake – participants aged under 65 years

Table S2 Dietary micronutrient intake – participants aged 65 and older

Table S3 Odds of low micronutrient intake associated with social isolation and loneliness: Participants aged less than 65 years

Table S4 Odds of low micronutrient intake associated with social isolation and loneliness: Participants aged 65 and older

Table S5 Odds of low micronutrient intake associated with social isolation and loneliness, excluding individuals taking relevant supplements

Table S6 Odds of low micronutrient intake associated with social isolation and loneliness: Fully adjusted models including impaired activities of daily living as a covariate

Table S1 Dietary micronutrient intake – participants aged under 65 years

Micronutrient	Estimated daily intake Mean ± SD	Recommended dietary intake threshold	Sufficient N (%)	Low N (%)	Supplements N (%)
Calcium (mg/day)	890.1 ± 316.0	700 mg/day	829 (70.9%)	341 (29.1%)	67 (5.8%)
Iron (mg/day)	12.4 ± 4.21	8.7 mg/day	944 (80.7%)	226 (19.3%)	51 (4.4%)
Magnesium (mg/day)	343.2 ± 101.1<br>306.5 ± 92.3	300 mg/day (men)<br>270 mg/day (women)	750 (64.1%)	420 (35.9%)	121 (10.3%)
Potassium (mg/day)	3450.0 ± 1061.3	3500 mg/day	530 (45.3%)	640 (54.7%)	102 (8.7%)
Vitamin B6 (mg/day)	2.13 ± 0.68<br>1.97 ± 0.67	1.0 mg/day (men)<br>0.8 mg/day (women)	1031 (88.1%)	139 (11.9%)	115 (9.8%)
Folate (µg/day)	272.8 ± 106.0	200 µg/day	889 (76.0%)	281 (24.0%)	110 (9.4%)
Vitamin B12 (µg/day)	5.96 ± 4.06	1.5 (µg/day)	1110 (94.9%)	60 (5.1%)	120 (10.2%)
Vitamin C (mg/day)	128.6 ± 99.2	40 mg/day	1025 (87.6%)	145 (12.4%)	150 (12.8%)
Vitamin E (mg/day)	8.54 ± 4.20<br>8.60 ± 3.79	4.0 mg/day (men)<br>3.0 mg/day (women)	1093 (93.4%)	77 (6.6%)	104 (8.8%)

Table S2 **Dietary micronutrient intake – participants aged 65 and older**

Micronutrient	Estimated daily intake Mean ± SD	Recommended daily intake threshold	Sufficient N (%)	Low N (%)	Supplements N (%)
Calcium (mg/day)	932.6 ± 316.9	700 mg/day	1945 (76.5%)	598 (23.5%)	202 (8.0%)
Iron (mg/day)	12.8 ± 4.06	8.7 mg/day	2168 (85.3%)	375 (14.7%)	106 (4.2%)
Magnesium (mg/day)	339.0 ± 98.1<br>306.4 ± 92.1	300 mg/day (men)<br>270 mg/day (women)	1605 (63.1%)	938 (36.9%)	267 (10.5%)
Potassium (mg/day)	3538.9 ± 1066.4	3500 mg/day	1209 (47.4%)	1334 (52.5%)	222 (8.7%)
Vitamin B6 (mg/day)	2.18 ± 0.72<br>1.97 ± 0.67	1.0 mg/day (men)<br>0.8 mg/day (women)	2263 (89.0%)	280 (11.0%)	262 (10.4%)
Folate (µg/day)	286.8 ± 111.0	200 µg/day	1995 (78.5%)	548 (21.5%)	257 (10.1%)
Vitamin B12 (µg/day)	6.47 ± 3.96	1.5 (µg/day)	2470 (97.1%)	73 (2.9%)	289 (11.4%)
Vitamin C (mg/day)	133.6 ± 92.3	40 mg/day	2282 (89.7%)	261 (10.3%)	358 (14.0%)
Vitamin E (mg/day)	8.87 ± 4.00<br>8.80 ± 4.03	4.0 mg/day (men)<br>3.0 mg/day (women)	2407 (94.7%)	136 (5.3%)	247 (9.7%)

Table S3 **Odds of low micronutrient intake associated with social isolation and loneliness Participants aged less than 65 years**

	Social isolation				Loneliness			
	Adjusted for age, gender and total energy intake		Fully adjusted[1]		Adjusted for age, gender and total energy intake		Fully adjusted[1]	
	OR (95%CI)	P	OR (95%CI)	P	OR (95%CI)	P	OR (95%CI)	P
Calcium	0.999 (0.835-1.195)	0.99	0.942 (0.783-1.135)	0.53	0.988 (0.893-1.093)	0.82	0.930 (0.835-1.037)	0.19
Iron	1.395 (1.126-1.728)	0.002	1.259 (1.006-1.576)	0.044	1.133 (1.008-1.273)	0.036	1.105 (0.975-1.254)	0.12
Magnesium	1.233 (1.019-1.491)	0.031	1.092 (0.895-1.3332	0.39	1.081 (0.972-1.202)	0.15	1.035 (0.922-1.163)	0.56
Potassium	1.270 (1.065-1.513)	0.008	1.222 (1.019-1.465)	0.030	1.123 (1.015-1.243)	0.025	1.072 (0.961-1.195)	0.21
Vitamin B6	1.158 (0.926-1.448)	0.20	1.099 (0.869-1.390)	0.43	1.079 (0.952-1.223)	0.24	1.050 (0.917-1.203)	0.48
Folate	1.255 (1.056-1.492)	0.010	1.162 (0.970-1.392)	0.10	1.117 (1.015-1.229)	0.023	1.103 (0.994-1.224)	0.065
Vitamin B12	0.934 (0.680-1.282)	0.67	0.911 (0.654-1.268)	0.58	1.081 (0.916-1.275)	0.36	1.021 (0.851-1.224)	0.83
Vitamin C	1.520 (1.251-1.847)	<0.0001	1.347 (1.098-1.652)	0.004	1.188 (1.068-1.320)	0.001	1.157 (1.029-1.300)	0.015
Vitamin E	1.524 (1.150-2.021)	0.003	1.347 (0.997-1.820)	0.053	1.136 (0.965-1.337)	0.12	1.055 (0.882-1.262)	0.56

[1]Adjusted for age, gender, ethnicity, education, total energy intake, marital status, smoking and physical activity

Table S4 **Odds of low micronutrient intake associated with social isolation and loneliness**
Participants aged 65 and older

	**Social isolation**				**Loneliness**			
	Adjusted for age, gender and total energy intake		Fully adjusted[1]		Adjusted for age, gender and total energy intake		Fully adjusted[1]	
	OR (95%CI)	P	OR (95%CI)	P	OR (95%CI)	P	OR (95%CI)	P
Calcium	1.137 (1.005-1.286)	0.042	1.111 (0.981-1.260)	0.98	0.977 (0.902-1.058)	0.57	0.963 (0.886-1.047)	0.38
Iron	1.147 (0.980-1.343)	0.087	1.087 (0.926-1.277)	0.31	1.063 (0.964-1.172)	0.22	1.001 (0.903-1.109)	0.99
Magnesium	1.264 (1.118-1.430)	<0.0001	1.177 (1.037-1.336)	0.012	1.122 (1.018-1.213)	0.004	1.078 (0.992-1.171)	0.076
Potassium	1.248 (1.109-1.404)	<0.001	1.191 (1.057-1.343)	0.004	1.104 (1.023-1.191)	0.011	1.064 (0.982-1.152)	0.13
Vitamin B6	1.376 (1.180-1.605)	<0.0001	1.342 (1.148-1.569)	<0.001	1.067 (0.966-1.177)	0.20	1.038 (0.936-1.151)	0.48
Folate	1.285 (1.136-1.454)	<0.0001	1.226 (1.082-1.390)	0.001	1.073 (0.993-1.169)	0.073	1.037 (0.956-1.124)	0.38
Vitamin B12	0.952 (0.720-1.260)	0.73	0.890 (0.663-1.194)	0.44	1.020 (0.861-1.209)	0.82	0.979 (0.816-1.173)	0.82
Vitamin C	1.264 (1.093-1.461)	0.002	1.187 (1.023-1.378)	0.024	1.066 (0.970-1.171)	0.19	0.999 (0.904-1.105)	0.99
Vitamin E	1.065 (0.861-1.318)	0.61	1.007 (0.809-1.253)	0.95	1.158 (1.013-1.324)	0.031	1.131 (0.980-1.305)	0.092

[1]Adjusted for age, gender, ethnicity, education, total energy intake, marital status, smoking and physical activity

Table S5 **Odds of low micronutrient intake associated with social isolation and loneliness, excluding individuals taking relevant supplements**

	**Social isolation**				**Loneliness**			
	Adjusted for age, gender and total energy intake		Fully adjusted[1]		Adjusted for age, gender and total energy intake		Fully adjusted[1]	
	OR (95%CI)	P	OR (95%CI)	P	OR (95%CI)	P	OR (95%CI)	P
Calcium	1.112 (1.001-1.236)	0.048	1.079 (0.969-1.201)	0.16	0.975 (0.914-1.041)	0.45	0.946 (0.884-1.014)	0.12
Iron	1.235 (1.084-1.406)	<0.001	1.149 (1.005-1.313)	0.042	1.077 (0.997-1.163)	0.060	1.018 (0.938-1.104)	0.68
Magnesium	1.265 (1.134-1.411)	<0.0001	1.158 (1.034-1.297)	0.011	1.117 (1.046-1.193)	0<0.001	1.078 (1.005-1.157)	0.037
Potassium	1.256 (1.134-1.391)	<0.0001	1.196 (1.078-1.328)	<0.001	1.104 (1.036-1.176)	0.002	1.065 (0.996-1.139)	0.065
Vitamin B6	1.281 (1.121-1.463)	<0.0001	1.238 (1.081-1.419)	0.002	1.058 (0.975-1.147)	0.18	1.024 (0.938-1.117)	0.60
Folate	1.287 (1.157-1.431)	<0.0001	1.216 (1.091-1.335)	<0.001	1.098 (1.030-1.169)	0.004	1.066 (0.997-1.140)	0.063
Vitamin B12	0.918 (0.725-1.163)	0.48	0.876 (0.685-1.121)	0.29	1.034 (0.907-1.180)	0.62	1.001 (0.869-1.154)	0.98
Vitamin C	1.336 (1.181-1.513)	<0.0001	1.224 (1.077-1.391)	0.002	1.089 (1.009-1.174)	0.028	1.025 (0.945-1.112)	0.56
Vitamin E	1.172 (0.979-1.403)	0.083	1.087 (0.902-1.312)	0.38	1.181 (1.061-1.315)	0.002	1.123 (0.999-1.263)	0.053

[1]Adjusted for age, gender, ethnicity, education, total energy intake, marital status, smoking and physical activity

Table S6 **Odds of low micronutrient intake associated with social isolation and loneliness**
Fully adjusted models including impaired activities of daily living as a covariate

	**Social isolation**		**Loneliness**	
	Adjusted OR (95%CI)	P	Adjusted OR (95%CI)	P
Calcium	1.061 (0.957-1.177)	0.26	0.956 (0.895-1.022)	0.19
Iron	1.137 (0.999-1.295)	0.052	1.025 (0.946-1.111)	0.54
Magnesium	1.150 (1.034-1.279)	0.010	1.058 (0.989-1.133)	0.10
Potassium	1.199 (1.086-1.325)	<0.001	1.067 (1.000 – 1.138)	0.050
Vitamin B6	1.265 (1.111-1.439)	<0.001	1.044 (0.960-1.134)	0.32
Folate	1.208 (1.090-1.338)	<0.001	1.048 (0.983-1.118)	0.15
Vitamin B12	0.912 (0.733-1.134)	0.41	0.994 (0.847-1.131)	0.93
Vitamin C	1.237 (1.098-1.394)	<0.001	1.056 (0.978-1.140)	0.16
Vitamin E	1.110 (0.932-1.3222	0.24	1.098 (0.981-1.229)	0.103

[1]Adjusted for age, gender, ethnicity, education, total energy intake, marital status, smoking, physical activity, and impaired activities of daily living
